# An exposure-based implementation strategy to decrease clinician anxiety about implementing suicide prevention evidence-based practices: protocol for development and pilot testing (Project CALMER)

**DOI:** 10.1186/s43058-023-00530-3

**Published:** 2023-11-24

**Authors:** Emily M. Becker-Haimes, Megan Brady, Jesslyn Jamison, Shari Jager-Hyman, Megan E. Reilly, Esha Patel, Gregory K. Brown, David S. Mandell, Maria A. Oquendo

**Affiliations:** 1 Department of Psychiatry, University of Pennsylvania Perelman School of Medicine, 3535 Market Street, 3 Rd floor, Philadelphia, PA 19104 USA; 2 Hall Mercer Community Mental Health, University of Pennsylvania Health System, Philadelphia, USA

**Keywords:** Clinician anxiety, Implementation, Evidence-based implementation strategy, Exposure therapy, Suicide prevention

## Abstract

**Background:**

Clinicians often report that their own anxiety and low self-efficacy inhibit their use of evidence-based suicide prevention practices, including gold-standard screening and brief interventions. Exposure therapy to reduce clinician maladaptive anxiety and bolster self-efficacy use is a compelling but untested approach to improving the implementation of suicide prevention evidence-based practices (EBPs). This project brings together an interdisciplinary team to leverage decades of research on behavior change from exposure theory to design and pilot test an exposure-based implementation strategy (EBIS) to target clinician anxiety to improve suicide prevention EBP implementation.

**Methods:**

We will develop, iteratively refine, and pilot test an EBIS paired with implementation as usual (IAU; didactic training and consultation) in preparation for a larger study of the effect of this strategy on reducing clinician anxiety, improving self-efficacy, and increasing use of the Columbia Suicide Severity Rating Scale and the Safety Planning Intervention in outpatient mental health settings. Aim 1 of this study is to use participatory design methods to develop and refine the EBIS in collaboration with a stakeholder advisory board. Aim 2 is to iteratively refine the EBIS with up to 15 clinicians in a pilot field test using rapid cycle prototyping. Aim 3 is to test the refined EBIS in a pilot implementation trial. Forty community mental health clinicians will be randomized 1:1 to receive either IAU or IAU + EBIS for 12 weeks. Our primary outcomes are EBIS acceptability and feasibility, measured through questionnaires, interviews, and recruitment and retention statistics. Secondary outcomes are the engagement of target implementation mechanisms (clinician anxiety and self-efficacy related to implementation) and preliminary effectiveness of EBIS on implementation outcomes (adoption and fidelity) assessed via mixed methods (questionnaires, chart-stimulated recall, observer-coded role plays, and interviews).

**Discussion:**

Outcomes from this study will yield insight into the feasibility and utility of directly targeting clinician anxiety and self-efficacy as mechanistic processes informing the implementation of suicide prevention EBPs. Results will inform a fully powered hybrid effectiveness-implementation trial to test EBIS’ effect on implementation and patient outcomes.

**Trial registration:**

Clinical Trials Registration Number: NCT05172609. Registered on 12/29/2021.

Contributions to the literature

• Clinician anxiety and self-efficacy are potential target mechanisms for implementation strategy design for promoting the use of evidence-based suicide prevention strategies.
• This study will be the first to apply established behavior change and emotion regulation techniques (i.e., exposure-based strategies) to directly target these theorized mechanisms in service of improving implementation outcomes for suicide prevention.
• Results of this study will provide data on the feasibility, acceptability, and engagement of target mechanisms of an exposure-based implementation strategy (EBIS) to increase gold-standard suicide prevention practice use in preparation for larger confirmatory trials.

## Background

Designing and testing scalable implementation strategies to increase the use of evidence-based screening [1], assessment [2], and brief suicide prevention interventions [3, 4] are critical next steps for advancing suicide prevention efforts. There are about 800,000 deaths by suicide globally annually [5]. Numerous studies demonstrate the effectiveness of evidence-based practices (EBPs) for suicide screening, assessment, and intervention (or “SSAIs”) with individuals at risk for suicide [6]. Despite the evidence supporting SSAIs like the Columbia Suicide Severity Rating Scale [C-SSRS] for screening and assessment [1] and the Safety Planning Intervention [SPI] for brief intervention [3], they remain underused in routine clinical settings.

Leading implementation scientists have argued that implementation strategies that address specific facilitators and barriers to EBP delivery will be more successful than general strategies [7–10]. Choosing effective strategies requires identifying the specific mechanisms that affect implementation success [11]. To date, implementation strategies to improve the delivery of suicide prevention EBPs have not majorly attempted to target specific mechanisms [10].

**Fig. 1:**
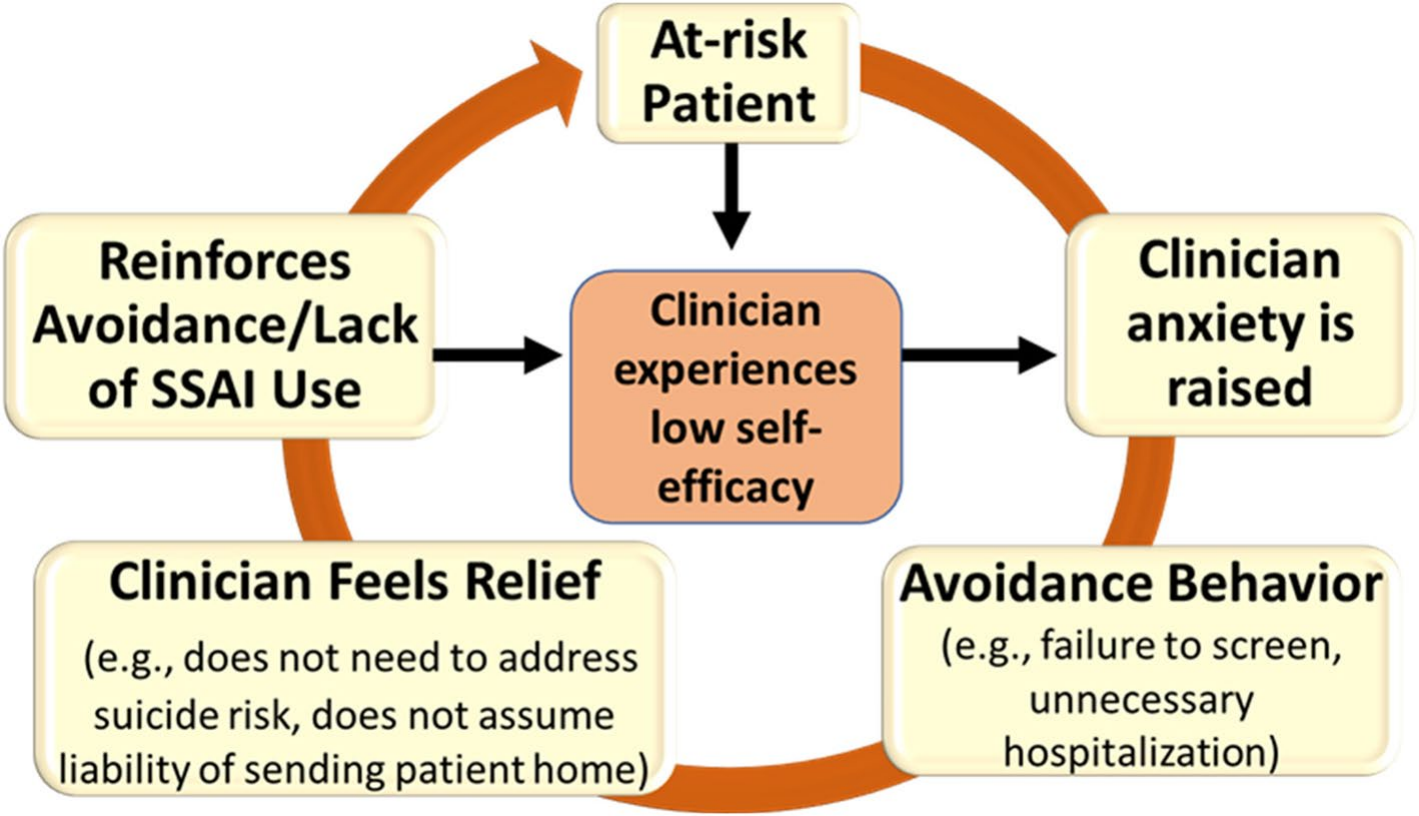
Conceptual model of maladaptive anxious avoidance applied to suicide prevention EBPs, adapted from Becker-Haimes et al. ([12]; permission given)

One possible set of mechanisms that inhibit the uptake of suicide prevention EBPs is clinician anxiety and low self-efficacy related to practice use [12]. Clinicians can experience intense anxiety when patients endorse suicide risk [13] due to fear of liability, fear of miscalculating patient risk, uncertainty about how to intervene, and fear of having inadequate time to intervene [14]. Anticipatory anxiety about suicide risk paradoxically can lead to inadequate assessment or avoidance of screening entirely [15–17], consistent with maladaptive avoidance processes commonly associated with anxiety (see Fig. 1).

A robust qualitative literature delineates how clinicians’ fear and anxiety about their ability to support patients in managing suicide risk can lead to them avoiding EBPs [18], sometimes with detrimental consequences [17]. Emerging quantitative data support these findings. For example, a survey of 300 practicing mental health clinicians found that nearly half endorsed high anxiety about patient death by suicide, even though most of the sample reported high to very high knowledge of how to work with suicidal patients [19]. Other studies find that many providers fear, wrongly, that asking about suicide will increase patient suicide risk [20], which leads them to avoid using SSAIs or employ “shut down” language that minimizes the potential for actual suicidal risk to be disclosed [21]. Clinician discomfort working with patients at risk for suicide also negatively affects patient experience and their likelihood of disclosure, due to patient perceptions that their clinician would be concerned about liability and because they were worried about the clinician’s adverse emotional response to disclosures [22, 23].

High anxiety about SSAIs also can be driven by low self-efficacy or lack of confidence in one’s ability to effectively intervene to mitigate suicide risk [2, 19]. A recent review noted that self-efficacy potentially mediates the relationship between training and implementation outcomes [24]. Higher self-efficacy to intervene with patients at risk for suicide is an important independent predictor of SSAI use [25], perhaps because SSAI delivery is complex and can require intensive support to implement correctly [26]. For example, LoParo and colleagues [15] found a link between greater self-efficacy in SSAI skills and more use of EBPs for SSAIs in a sample of 137 community mental health clinicians.

Theoretical models of maladaptive anxiety posit that low self-efficacy can increase anticipatory anxiety and thus the likelihood of maladaptive avoidance [27]. For example, low clinician self-efficacy for SSAI delivery can prompt decisions to hospitalize patients out of fear that either a patient will attempt suicide or that there will be legal repercussions of inaction, rather than because of a patient’s true risk [28–30]. These unnecessary hospitalizations can have iatrogenic effects and are costly [31], yet likely reinforce clinicians’ beliefs that hospitalizing a client is the safest path forward, as the clinician does not experience sending a high-risk patient home with a safety plan in place where safety is not guaranteed. This perpetuates the likelihood that clinicians will continue to experience anxiety about working with individuals at risk for suicide and continue to over-refer to crisis centers.

To our knowledge, no efforts have targeted clinician anxiety about SSAI use other than by offering training [19], which many studies have established as insufficient to change behavior [32, 33]. Guided by a conceptual model delineating how clinician anxious avoidance drives suboptimal implementation [12] (see Fig. 1), we will develop and test an implementation strategy grounded in principles of learning theory and exposure therapy*.* Exposure therapy is based on the established theory that anxiety is maintained and worsened through avoidance of feared stimuli [34]. It involves guiding individuals to gradually confront and increase their tolerance of stimuli [35] in four phases: (1) psychoeducation about how maladaptive anxiety influences behavior; (2) assessing individuals’ feared outcomes and ranking ease of possible practice scenarios; (3) repeated facilitated practice in facing fears followed by cognitive debriefing; and (4) independent application to real-life (“relapse prevention”) [36]. It is the key cognitive-behavioral treatment ingredient leading to reduced maladaptive anxiety and increased self-efficacy to engage with feared scenarios [37–40].

**Fig. 2:**
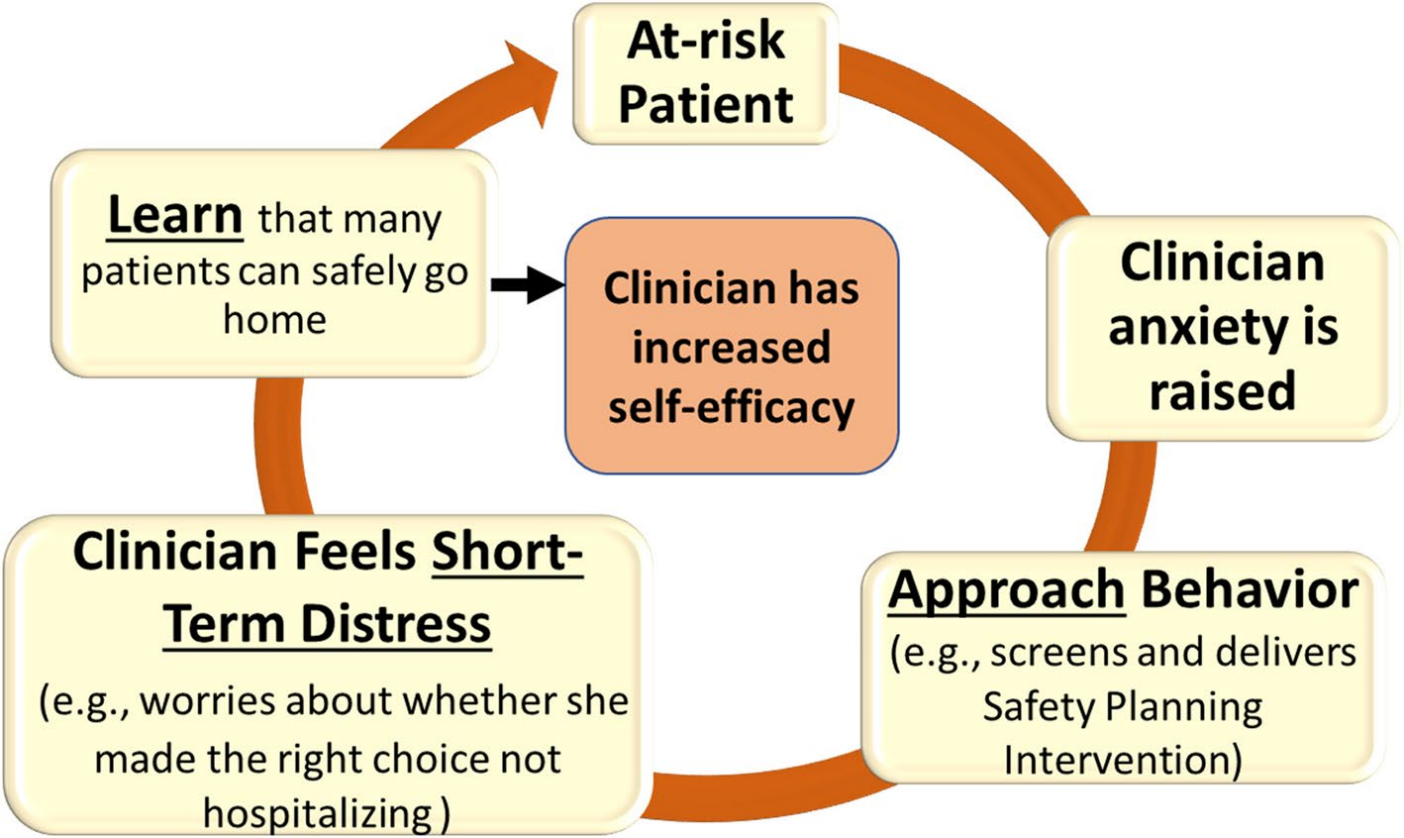
Facilitating a cycle of approach for SSAI use

We will design an exposure-based implementation strategy (EBIS) to augment traditional training approaches. The EBIS is intended to directly target and mitigate clinician anxiety related to working with individuals at risk for suicide and bolster clinician self-efficacy to implement SSAIs with their patient population. This approach has been used to mitigate clinician anxiety about delivering exposure therapy itself to anxious patients, with pilot work showing promise for improving exposure therapy use [36], but has not been used to support SSAI implementation, which may be more challenging for clinicians. Figure 2 illustrates how exposure therapy can address target mechanisms.

This study (Clinician Affect Labeling and Management through Exposure Research; Project CALMER) is one of three exploratory projects through the NIMH-funded Penn Innovation in Suicide Prevention Implementation Research (INSPIRE) Center [41], which brings together experts in psychology, implementation science, health economics, machine learning, health information technology, and psychiatry to address suicide prevention. Project CALMER aims to increase clinicians’ high-fidelity implementation of SSAI by augmenting training and consultation with brief graduated exposure therapy that combines imaginal and in vivo exposures [42] with standardized patients. Specific aims are to:

- Aim 1: Using participatory design methods, develop the EBIS to reduce clinician anxiety and bolster self-efficacy to deliver SSAI by leveraging exposure-based therapies to manage core clinician fears and anxiety related to suicide in high-risk patient encounters.
- Aim 2: Iteratively refine the EBIS through pilot field-testing using rapid cycle prototyping with up to 20 clinicians. Participants will receive EBIS training and provide qualitative feedback to inform subsequent refinements.
- Aim 3: Test acceptability, feasibility, and preliminary effectiveness of the EBIS compared with implementation as usual (IAU) to target SSAI implementation mechanisms and outcomes in a pilot randomized trial with 40 clinicians. Exploratory analyses will examine the impact of both conditions on putative implementation mechanisms (clinician anxiety, self-efficacy) and outcomes (SSAI adoption, fidelity). We hypothesize EBIS will be an acceptable and feasible implementation strategy and that it will engage target implementation mechanisms of clinician anxiety and self-efficacy better than IAU and improve rates of SSAI use relative to IAU.

## Methods/design

### Study setting

All research activities will take place in the City of Philadelphia. The City of Philadelphia Department of Behavioral Health and Intellectual disAbility Services has prioritized suicide prevention across behavioral health service settings. The City of Philadelphia has selected two suicide prevention EBPs that are of focus in this study: the Columbia Suicide Severity Rating Scale (C-SSRS) and the Safety Planning Intervention (SPI) [3, 43]. The C-SSRS is an assessment tool designed to guide clinicians in assessing individual suicide risk; it includes direct questions clinicians ask to assess past and current suicidal ideation, intent, plan, and behaviors, as well as non-suicidal-self injury. The C-SSRS is well-established and freely available, making it attractive for widespread implementation. The SPI is a brief, single-session intervention shown to reduce the risk of suicide attempts. Clinicians work together with at-risk patients to identify warning signs of future suicidal crises and identify a series of actionable steps patients can take to minimize suicide risk. The SPI has a growing evidence base in support of its effectiveness across settings [3, 44, 45]. Despite this, our pilot work demonstrated that there is variability in the extent to which these EBPs are routinely implemented as intended in the outpatient setting [13]. Specific outpatient sites for participation will be selected in collaboration with our advisory board.

### Advisory board

We will conduct the study in collaboration with a diverse set of stakeholders from the Philadelphia region, including mental healthcare providers (e.g., practicing clinicians, medical directors), policy advisors (e.g., members of city government), and community organizations (e.g., leadership from organizations that support families who have experienced suicide loss). We will meet with stakeholders primarily in the first year of the project during the development stages and keep them closely apprised throughout the trial period. Stakeholders will be compensated $50 for each hour of their time.

### Conceptual frameworks

First, our conceptual framework of clinician maladaptive anxious avoidance (Figs. 1 and 2) supports our selection of implementation mechanisms for clinician anxiety and self-efficacy, which we hope to target via the EBIS. Second, our measurement battery is guided by the conceptual model employed by the larger Penn INSPIRE Center that integrates principles of contextual determinants of implementation (e.g., organizational policies and procedures) [46] and behavior change theory (e.g., intentions and their determinants, including norms, attitudes, self-efficacy, knowledge, and beliefs) [47–50].

### Aims 1 and 2: EBIS strategy development and refinement

Aims 1 and 2 will be completed concurrently. We will develop a prototype of EBIS based on several key components of exposure treatment. EBIS is informed by the latest science in exposure theory [35], borrowing heavily from brief exposure-based treatments that can be delivered in a single session [51–53]. We anticipate that EBIS will occur in four phases that map on to standard exposure therapy practice for patients with anxiety disorders: psychoeducation, assessment, practice, and relapse prevention; however, the final version will be based on Aim 1. Table 1 presents each anticipated component; each is also described more below.

**Table 1:** Anticipated EBIS components and rationale

EBIS component	Rationale
1. Psychoeducation	Obtain clinician buy-in, explain how anxiety can interfere with suicide EBP delivery, normalize clinician anxious experience, increase motivation to build insight into one’s own anxiety
2. Assessment/hierarchy building	Identify tailored exposure practice targets that best match an individual clinician’s fears, continue to build insight into one’s own anxiety and how it may influence clinical practice
3. Exposure practice	Provide exposure to feared outcomes to foster clinician self-efficacy in managing high-risk patients, facilitate clinician practice managing anxiety in high-risk encounters
4. Relapse/prevention application to clinical practice	Transition “learning” about one’s ability to manage high-risk encounters to clinical practice within ongoing consultation; continue to use an exposure frame to support implementation

#### Psychoeducation

In exposure therapy, psychoeducation is a critical step toward obtaining buy-in to engage in exposure practice. Typically, psychoeducation includes information about both adaptive and maladaptive anxiety. We will provide brief information about the role of anxiety in SSAI delivery, explain the “cycle of avoidance” that can occur when one experiences anxiety related to patient suicide risk [12], and discuss clinical implications of clinician avoidance of SSAI delivery. We will present common ways that anxiety can present in clinicians specifically as it relates to suicide prevention work and encourage clinicians to reflect on the ways in which anxiety may be likely to present for them.

#### Assessment/hierarchy-building

We anticipate using the “post-it method” in a group format for assessment and hierarchy-building. Clinicians will be presented with 8–12 flashcards that delineate various fears they may endorse about interacting with a suicidal patient that range in intensity (e.g., “patient endorses passive suicidal ideation” to “patient endorses a suicide plan”) and fears related to outcomes of intervention (e.g., “patient completes SPI but attempts suicide after leaving clinic,” “I am sued because my patient attempted suicide”). Clinicians will rank-order each fear with respect to how anxiety-provoking it is and rate their anticipated anxiety using the Subjective Units of Distress Scale (SUDS) for each scenario. We will select up to three of the most-feared scenarios for exposure practice at the conclusion of didactic training.

#### Guided practice: in vivo and imaginal exposure [54]

Clinicians will engage in either in vivo practice or imaginal exposure with a trained actor (research assistant; RA) based on feared scenarios selected in the step above. Consistent with standard practices [54], RAs or other members of the research team will guide clinicians to (1) identify core fears and anticipated anxiety via SUDS ratings, (2) engage in targeted practice to violate assumptions of core fears and track SUDS changes, and (3) engage in targeted cognitive debriefing to enhance coping self-efficacy. RAs will be guided by a previously developed “exposure checklist” that will be adapted for the EBIS to ensure adherence to the EBIS and will be closely supervised by licensed clinicians.

#### Relapse prevention/application to clinical practice

At the end of initial training, clinicians will be guided by the trainer(s) to summarize their experience and set intentions for managing anxiety they may experience in future clinical practice. Additional content in this EBIS domain will be embedded into follow-up consultation calls led by training staff and will include targeted discussion of clinician anxiety and avoidance alongside standard barriers in clinical practice to facilitate the maintenance of gains.

We will present the initial EBIS prototype to our Advisory Board for feedback and refinement, using a modified process of co-design [55, 56]. Meetings will be facilitated by project leads to encourage all stakeholders to provide feedback on general components of the strategy and how to structure each component for optimal results. Questions posed to the Board for EBIS components will be both general (e.g., “Tell us your thoughts on the psychoeducation component?”, “What exposure tasks do you think will be most important?”) and specific (e.g., “How can we make the psychoeducation component more engaging?”, “How can we optimize imaginal exposure to a feared outcome of being sued?”) with the goal of designing for acceptability and feasibility across diverse settings. We will integrate all feedback into a revised prototype that we will present to the Advisory Board in a second meeting.

After the second Board meeting, we will further refine EBIS in a pilot field test using rapid cycle prototyping with up to 20 mental health clinicians recruited from local outpatient community clinics.

#### Prototyping procedures

In each round of prototyping, we will recruit clinicians to participate in pilot EBIS versions. Clinicians will be recruited from local community mental health clinics; before recruiting, we will reach out to clinic leadership for approval to contact clinicians at each location. Clinicians will be eligible if they are practicing mental health clinicians in the City of Philadelphia who provide direct mental health services to a treatment-seeking population. Clinician participants will be excluded if they do not see any patients that are at risk for suicide (e.g., they screen out all high-risk patients for their individual practice). Participants will not be excluded based on sex, demographics, and/or experience. All participants will complete informed consent prior to study procedures via a scheduled phone or videoconference meeting with a trained research staff member.

We anticipate conducting 3–4 rounds of prototyping. In each round, consenting clinicians participating will receive some or all of the 4–6 h of training and EBIS content (exact length dependent on the EBIS design) either virtually or in person; full training content will include all gold-standard didactic and experiential practice typically included in trainings for the C-SSRS and the SPI, with the EBIS incorporated. Pilot trainings will be conducted by content experts on the research team and take place in small groups of 2–5 clinicians. Given the training length, it is anticipated training will occur up to over 2–3 days. We will video record all trainings to allow for quality checks. Video recordings will be reviewed by research staff after each training to identify “stuck points” (e.g., portions of the training that do not seem to flow smoothly) and areas where a clinician expresses confusion or exhibits other reactions to the EBIS components. These components will be flagged for review, discussion, and potential refinement between cycles.

Within approximately 1 week of the training, participants will complete a qualitative interview (45–60 min) with a member of the study team. Interviews will be structured around the clinician’s experience of and feedback on the strategy. Specific constructs include EBIS acceptability and feasibility and their perceived anxiety and self-efficacy about delivering the C-SSRS and SPI. We will query clinicians about the organizational policies and procedures they believe should be in place for the EBIS to be successful; information from this component of the qualitative interview will inform the development of an organizational policies checklist to be included in the Aim 3 trial. Clinicians will be provided with continuing education (CE) credits at no cost to them for participating and $75 for completing the follow-up feedback interview.

After each prototyping round (~ 2–5 per round), we will present information gathered, challenges identified, and suggested refinements to the EBIS to our Advisory Board to inform iterative changes. This process will repeat until we reach thematic saturation and have a version of the EBIS ready for testing in Aim 3.

### Aim 3: Pilot randomized clinical trial: implementation as usual (IAU) vs. IAU + EBIS

Aim 3 will test the acceptability, feasibility, and preliminary effectiveness of IAU + EBIS to target implementation mechanisms and outcomes relative to gold-standard IAU in a randomized pilot trial.

#### Aim 3 procedures

We anticipate partnering with 5 outpatient clinics to enroll 40 clinicians in the pilot trial; additional clinics will be recruited to participate as needed. Inclusion and exclusion criteria are identical to Aim 2. Sample size was selected in anticipation of a small to medium effect size with respect to anxiety reduction and increases in self-efficacy (target mechanisms) between the two treatment arms, using established rules of thumb for designing pilot clinical trials [57]. Interested clinicians will complete informed consent prior to study procedures via a scheduled phone, in-person, or videoconference meeting with a trained research staff member. Clinicians who agree to participate will be randomized 1:1 by senior study staff into one of two arms: IAU or the EBIS condition (IAU + EBIS). Participants will be aware of group assignment and informed that participation is voluntary and that they can discontinue participation at any time.

#### IAU

Gold-standard IAU typically comprises pre-implementation preparation, didactic training, knowledge tests, experiential role plays, ongoing expert consultation, and providing completion certificates to clinicians who complete all training and consultation activities. Pre-implementation preparation will include providing materials (e.g., SPI manual [3], instructions [58], and forms). Didactic training will occur in two parts: (1) C-SSRS screening and assessment and (2) SPI use. Part one will consist of materials we previously developed based on community clinician feedback. Part two will follow established SPI guidelines, including didactic training about SPI rationale and evidence base and experiential practice. After training, clinicians will receive 6 biweekly expert consultation sessions to discuss implementation barriers and practice through role plays.

#### EBIS

Clinicians randomized to EBIS will receive all IAU elements outlined above plus the EBIS refined in Aims 1 and 2.

Clinical trial data will include clinician responses to self-report questionnaires, chart-stimulated recall, semi-structured interviews, and fidelity role-plays with research staff. Recruitment and retention statistics will capture feasibility metrics. No identifiable data about patients will be collected. All data will be used exclusively for research purposes and administered at the following time points: T1 = baseline/pretraining; T2 = post-training; T3 = 2-week follow-up; T4 = 12-week follow-up. Between T1 and T2, clinicians will complete 4–6 h of suicide prevention training aligned with their assigned training condition for CE credit. Between T2 and T4, clinicians in each condition will receive a biweekly consultation protocol specific to their condition for 12 weeks. Clinicians will be compensated $50 for T1 measures, $25 for T2 measures, $100 for T3, and $125 for T4. This compensation protocol was selected to promote participant retention based on past experiences conducting community-based implementation trials.

Table 2 shows the assessment battery and timeline for Aim 3. Primary trial outcomes include EBIS acceptability and feasibility, measured both quantitatively and qualitatively. Secondary outcomes include target implementation mechanisms of clinician anxiety and self-efficacy, as well as exploratory implementation outcomes. Specific measures are described below. All measures will be administered to all clinicians enrolled in the trial.

**Table 2:** Study timeline for Aim 3 participant procedures

Construct assessed	Measures	**Study period time points**				
		Enrollment	Time 1 (pre-training)	Time 2 (post-training)	Time 3 (2-week follow-up)	Time 4 (12-week follow-up)
**Enrollment:**						
Consent to contact	n/a	x				
Informed consent	n/a	x				
**Primary outcomes:**						
EBIS acceptability	Acceptability of Intervention Measure			X		X
EBIS feasibility	Feasibility of Intervention Measure			X		X
**Target implementation mechanisms:**						
Clinician anxiety about SSAIs	Subjective Units of Distress Scale Suicidal Patient Comfort Survey		X	X	X	X
Clinician anxiety	Anxiety Sensitivity Index-3		X			
Clinician self-efficacy	Established behavioral science question stems		X	X	X	X
**Implementation outcomes:**						
SSAI use (C-SSRS, SPI)	Chart-Stimulated Recall		X		X	X
SPI fidelity	Role play coded with SPIRS				X	X
**Additional implementation mechanisms of interest:**						
SSAI knowledge	Self-Perceived Knowledge About Suicide Scale		X	X	X	X
SSAI beliefs	Clinician Attitudes Toward Safety Planning		X	X	X	X
SSAI attitudes	Established behavioral science question stems		X	X	X	X
SSAI norms	Established behavioral science question stems		X	X	X	X
SSAI intentions	Established behavioral science question stems		X	X	X	X
Organizational policies and procedures	Checklist developed under Aim 2 activities		X			
Contextual factors	Qualitative Interview					X
**Additional constructs of interest**						
Demographics	Demographics & Background Questionnaire		X			
Prior training experiences	Demographics & Background Questionnaire		X			

*Acceptability:* At T2 and T4, we will administer the Acceptability of Intervention Measure (AIM), a 4-item, psychometrically validated measure that measures the extent to which stakeholders believe an implementation strategy (in this case, IAU or EBIS) is acceptable [59]. Qualitative questions (see below) also will assess the perceived acceptability of assigned condition.

*Feasibility:* At T2 and T4, we will administer the Feasibility of Intervention Measure (FIM), which is a 4-item, psychometrically validated measure that measures the extent to which stakeholders perceive an implementation strategy (in this case, IAU or EBIS) is feasible [59]. We will rigorously track study attrition and note retention statistics at each timepoint. Qualitative questions (see below) will also assess the perceived feasibility of the assigned condition.

*Clinician anxiety:* We will measure anxiety specific to SSAI use and broad clinician anxiety. Our primary anxiety outcome will rely on the Subjective Units of Distress Scale (SUDS). The SUDS is a one-item, 10-point rating scale of perceived distress, commonly used to guide exposure therapy. This will be administered at T1–T4 separately for clinician anxiety about screening, assessment, and intervention. Clinicians also will complete the Suicidal Patient Comfort Survey, which is a brief, 5-item measure assessing clinician anxiety about interacting with and treating patients with suicidality. This will be administered at T1-T4. Finally, we will index broad clinician anxiety at baseline using the Anxiety Sensitivity Index (ASI-3). The ASI-3 is an 18-item measure that assesses an individual’s physical, cognitive, or social concerns about anxiety and its manifestations. This will be administered at T1.

*Clinician self-efficacy:* At all timepoints, we will measure clinician self-efficacy using validated question stems from behavioral science designed to measure a clinician’s own perceptions of whether they believe they have the skills and abilities to perform a task on a 7-point scale; these question stems are intended to be adapted to any behavior of interest [60]. Clinicians will report separately on their self-efficacy with respect to suicide screening, assessment, and intervention. Questions about screening apply to all patients; questions about assessment and intervention specifically reference clinician self-efficacy related to patients who screen positive for suicide risk.

*Clinician SSAI use:* We will use Chart Stimulated Recall (CSR), an established technique for examining clinician decision-making and clinical processes beyond what can be determined from chart review or self-report alone, to index SSAI use [61, 62]. We previously adapted CSR methods to measure SSAI use [13, 63]. A trained research team member will review the clinician’s caseload with them for the past clinic week and ask brief questions (no more than 5 min) related to the clinician’s suicide-related practices (e.g., Did you conduct a screen for suicide risk? How did you screen for risk? If risk was present, was a full C-SSRS/SPI administered?). This will be administered at T1, T3, and T4. No identifiable patient information is collected during the CSR. Clinicians are instructed to refrain from sharing any identifiable patient details.

*Clinician SSAI fidelity:* We will use a standardized role-play paradigm to index clinician SSAI fidelity at T3 and T4. Participants will receive a vignette and prepare for a 45–60-min role play, during which they will be asked to complete an SPI with a patient who was determined to be at risk for suicide following C-SSRS administration with a trained actor. This paradigm is a training method that provides a standardized opportunity for assessing skills and competencies [64]. The intent is to make the information in the clinical scenarios equally challenging in conducting the SPI among all standardized patients [65]. Role plays will be audio-recorded and coded for competence with the Safety Planning Intervention Rating Scale (SPIRS) [66]. Coders will be masked to the clinician study condition.

*Additional mechanisms of interest:* We will measure SSAI knowledge, beliefs, attitudes, norms, and intentions, and organizational policies and procedures that may facilitate implementation success. *Knowledge* will be indexed via a subscale of the Short Survey on Knowledge, Self-Confidence, and Attitudes Towards Suicidal Behavior to assess a clinician’s perceived knowledge about suicide and how to intervene. *Beliefs* will be assessed using an established 12-item questionnaire designed to assess clinician beliefs about the use of safety planning interventions in clinical practice with suicidal patients [67]. *Attitudes*, *norms*, and *intentions* will be measured via validated questions stemming from behavioral science using items on a 7-point scale [60]. For each of these constructs, we will ask clinicians to report separately on perceptions related to screening, assessment, and intervention, as appropriate. For attitudes, clinicians will rate items asking about the perceived clinical utility of each SSAI component from “strongly disagree” to “strongly agree”. For norms, clinicians will rate their perception of how other clinicians like them engage in SSAI use and the extent to which they feel their clinical supervisor will approve of them using each SSAI component from “strongly disagree” to “strongly agree”. We will measure intentions separately for each SSAI component. These all will be assessed at T1–T4. *Organizational policies and procedures* will be measured via a brief checklist developed following Aim 2 activities as described above at T1; additional contextual factors influencing implementation success will be measured qualitatively.

*Qualitative interview:* All clinicians will complete a three-section qualitative interview at T4. Section 1 will query about the acceptability of the implementation condition to which they were assigned (EBIS or IAU) and any barriers that arose in engaging with any study component. Section 2 will focus on how the implementation condition engaged target implementation mechanisms. The final section will be guided by the Consolidated Framework for Implementation Research [46] to query about barriers to SSAI use to identify contextual determinants not adequately addressed by EBIS.

*Clinician demographics:* At T1, we will administer a brief questionnaire about clinician demographic (e.g., age, sex) and professional characteristics (e.g., years of experience, theoretical orientation), and a question about the clinician’s personal experience with suicide (e.g., “Have you ever had a client die by suicide while under your care?”).

### Data analysis

Data screening and missing data analysis will be conducted in accordance with best practices. We will examine the psychometric properties of all scales used to assess constructs of interest (e.g., coefficient alpha) to confirm adequate psychometric performance. We will conduct analyses of baseline variables as a randomization check to ensure comparable baseline characteristics for clinicians in EBIS and IAU. Any differences will be controlled for in subsequent analyses.

#### Quantitative analyses

This pilot feasibility trial is not adequately powered to detect significant effects by design. Findings will provide key preliminary data to support the feasibility of EBIS and study procedures for a fully powered type 3 hybrid effectiveness-implementation trial. Qualitative and mixed methods analysis will supplement quantitative analyses to answer key questions of interest related to EBIS acceptability, feasibility, and target mechanism engagement.

*Hypothesis 1: EBIS will be an acceptable and feasible implementation strategy*. We will calculate descriptive statistics on the AIM and FIM measures and compare scores between EBIS and IAU. We will also evaluate EBIS feasibility by calculating the proportion of clinicians that are randomized to EBIS and complete all exposure tasks; we will compare study dropout rates and measure completion rates between conditions.

*Exploratory hypotheses 1 and 2: EBIS will engage target implementation mechanisms of clinician anxiety and self-efficacy better than IAU; clinicians randomized to EBIS will show improved SSAI adoption and fidelity relative to IAU*. We will examine the effect of condition over time on target mechanisms (anxiety and self-efficacy) and implementation outcomes (mean C-SSRS screening frequency across encounters, proportions of appropriate encounters in which clinicians conduct follow-up assessment with the C-SSRS and SPI use, as measured by CSR, and average SPI fidelity scores on role plays) using repeated measures analysis of covariance (ANCOVA), controlling for organization (given the small sample size and that we will recruit from only a small number of organizations for this pilot, we will not use multilevel analysis).

#### Qualitative and mixed-methods analyses

Qualitative analysis of interviews will complement quantitative data to examine: (1) the efficacy of EBIS to reduce clinician anxiety and bolster self-efficacy, (2) processes by which EBIS does or does not facilitate SSAI use, and (3) contextual factors in clinicians’ settings that are not addressed by EBIS. Interviews will be transcribed and analyzed via qualitative software, guided by an integrated approach which uses an inductive process of iterative coding to identify recurrent themes, categories, and relationships. We will develop a structured codebook and code for a priori attributes of interest (i.e., the extent to which EBIS engages our target mechanisms of anxiety and self-efficacy) and use modified grounded theory, which provides a systematic and rigorous approach to identifying codes and themes (e.g., to identify additional barriers that arise to SSAI use). Members of the research team will separately code three transcripts to develop an initial coding scheme, which will then be applied to subsequent transcripts. Code reliability will be rigorously monitored; if coder reliability drops below 0.85, each transcript will be coded by two separate raters, using a consensus procedure to reconcile discrepancies for all transcripts. We will employ mixed methods analyses in accordance with best practice recommendations [68–70].

#### Progression criteria

We will examine outcomes of acceptability and feasibility, and target engagement, to determine whether pilot trial results support the notion of progressing to a larger, confirmatory trial. For acceptability and feasibility, we will descriptively compare mean AIM and FIM scores between the two conditions; comparable acceptability and feasibility scores will be suggestive of the potential utility of a full trial. If EBIS scores are significantly lower than those within the IAU condition, this would raise concerns about the acceptability and feasibility of a larger trial. With respect to target engagement, we will be looking to see (1) greater pre- to post-reductions in clinician SUDS and greater pre- to post-increases in self-efficacy scores in the EBIS condition relative to IAU, and/or (2) anxiety reduction and self-efficacy to emerge as a theme in qualitative interviews more commonly and strongly in the EBIS condition relative to IAU.

### Data safety and monitoring

The larger NIMH-funded P50 Penn INSPIRE Center has convened a three-member DSMB that consists of an independent group of experts. This DSMB is charged with reviewing study data for data quality and integrity, adherence to the protocol, participant safety, study conduct and progress, and making determinations regarding study continuations, modifications, and suspensions/terminations for all INSPIRE projects, including this exploratory project. DSMB members are independent from any professional or financial conflict of interest with the research project or study investigators. The DSMB will meet at least once during the project.

## Discussion

This exploratory project will develop and evaluate the initial acceptability and feasibility of an EBIS specifically designed to reduce clinician anxiety and increase clinician self-efficacy related to the implementation of SSAIs. To our knowledge, this is the first implementation strategy to target these often-cited target mechanisms in suicide prevention implementation. This work is also responsive to the growing recognition of the importance of attending to clinician emotional reactivity within the context of implementation efforts [71] and explicitly identifying how clinician emotional responses may affect implementation processes [12]. Should data prove promising, we will test the refined EBIS in a large-scale confirmatory RCT and formally determine whether the EBIS reduces clinician anxiety and increases clinician self-efficacy and subsequent fidelity to SSAIs. In addition, SSAIs are just one example of evidence-based clinical practices that can be aversive to clinicians; future work will explore the extent to which an EBIS may support the implementation of other anxiety-provoking practices, such as time-out for disruptive behavior disorders [12].

### Design considerations

First, we considered utilizing a pilot type 3 hybrid-effectiveness implementation trial in Aim 3; we decided that collecting patient data in this small clinician sample was unlikely to be meaningful. However, we will explore how to collect patient data for future trials. Second, we considered directly observing clinicians delivering the SPI, rather than relying on standardized role plays. Role plays may lead to inflated fidelity, given that clinicians are not bound by the time constraints of real-world clinical practice; however, our prior work has demonstrated that role plays can be representative of direct observation [72]. We will address this concern by having clinicians rate how representative their role play felt of their clinical practice. Third, we considered working with a software company to develop exposure simulations with virtual or augmented reality. However, we thought it important to test the preliminary effectiveness of the exposure-based model before investing in expensive technological features.

### Future directions

Future directions include confirmatory trials to test EBIS effectiveness to engage target mechanisms and improve implementation and develop strategies for EBIS scale-up. Future work will include cost-effectiveness analysis of EBIS to support its adoption widely in community mental health and other clinical settings where providers are likely to encounter individuals at risk for suicide (e.g., schools, primary care) and other provider types who are asked to work with individuals at-risk for suicide (e.g., nurse practitioners). Future work also will include an examination of the timing of an EBIS in a clinician’s career trajectory to determine if there are optimal developmental stages (e.g., during graduate training, immediately post-graduation) that optimize response. We will also explore how an EBIS can support implementation of other EBPs that can elicit strong emotional reactions in clinicians that lead to underutilization of best practices (e.g., the use of time-out for disruptive behavior disorders).

## Conclusions

Outcomes will yield insight into the feasibility and importance of directly targeting clinician anxiety and self-efficacy as mechanistic processes informing suicide prevention EBP implementation. Should this study produce promising results, this work will lead to large-scale testing of an EBIS to promote the use of gold-standard suicide prevention EBPs across diverse contexts.

## Data Availability

Data sharing is not applicable to this article as no datasets were generated or analyzed during the current study. The final dataset will be shared to the NIMH Data Archive, consistent with the terms of the grant award.

## Abbreviations

AIM: Acceptability of Intervention Measure
ANCOVA: Repeated measures analysis of covariance
ASI-3: Anxiety Sensitivity Index
CALMER: Clinician Affect Labeling and Management through Exposure Research
C-SSRS: Columbia Suicide Severity Rating Scale
CSR: Chart Stimulated Recall
DSMB: Data and Safety Monitoring Board
EBIS: Exposure-based implementation strategy
EBPs: Evidence-based practices
FIM: Feasibility of Implementation Measure
IAU: Implementation as usual
INSPIRE: Innovation in Suicide Prevention Implementation Research
RA: Research assistant
RCT: Randomized controlled trial
SPI: Safety Planning Intervention
SPIRS: Safety Planning Intervention Rating Scale
SSAIs: Suicide screening, assessment, and intervention
SUDS: Subjective Units of Distress Scale
